# Sirtuin 1 and Autophagy Attenuate Cisplatin-Induced Hair Cell Death in the Mouse Cochlea and Zebrafish Lateral Line

**DOI:** 10.3389/fncel.2018.00515

**Published:** 2019-01-14

**Authors:** Jiaqi Pang, Hao Xiong, Ting Zhan, Gui Cheng, Haiying Jia, Yongyi Ye, Zhongwu Su, Hongyu Chen, Hanqing Lin, Lan Lai, Yongkang Ou, Yaodong Xu, Suijun Chen, Qiuhong Huang, Maojin Liang, Yuexin Cai, Xueyuan Zhang, Xiaoding Xu, Yiqing Zheng, Haidi Yang

**Affiliations:** ^1^Department of Otolaryngology, Sun Yat-sen Memorial Hospital, Sun Yat-sen University, Guangzhou, China; ^2^Department of Hearing and Speech Science, Xinhua College, Sun Yat-Sen University, Guangzhou, China; ^3^Guangdong Provincial Key Laboratory of Malignant Tumor Epigenetics and Gene Regulation, Medical Research Center, Sun Yat-sen Memorial Hospital, Sun Yat-sen University, Guangzhou, China; ^4^Department of Otolaryngology, The First Affiliated Hospital of Jinan University, Guangzhou, China; ^5^School of Public Health, Sun Yat-sen University, Guangzhou, China; ^6^RNA Biomedical Institute, Sun Yat-sen Memorial Hospital, Sun Yat-sen University, Guangzhou, China

**Keywords:** SIRT1, autophagy, cisplatin, cell death, ototoxicity

## Abstract

Cisplatin-induced ototoxicity is one of the major adverse effects in cisplatin chemotherapy, and hearing protective approaches are unavailable in clinical practice. Recent work unveiled a critical role of autophagy in cell survival in various types of hearing loss. Since the excessive activation of autophagy can contribute to apoptotic cell death, whether the activation of autophagy increases or decreases the rate of cell death in CDDP ototoxicity is still being debated. In this study, we showed that CDDP induced activation of autophagy in the auditory cell HEI-OC1 at the early stage. We then used rapamycin, an autophagy activator, to increase the autophagy activity, and found that the cell death significantly decreased after CDDP injury. In contrast, treatment with the autophagy inhibitor 3-methyladenine (3-MA) significantly increased cell death. In accordance with *in vitro* results, rapamycin alleviated CDDP-induced death of hair cells in zebrafish lateral line and cochlear hair cells in mice. Notably, we found that CDDP-induced increase of Sirtuin 1 (SIRT1) in the HEI-OC1 cells modulated the autophagy function. The specific SIRT1 activator SRT1720 could successfully protect against CDDP-induced cell loss in HEI-OC1 cells, zebrafish lateral line, and mice cochlea. These findings suggest that SIRT1 and autophagy activation can be suggested as potential therapeutic strategies for the treatment of CDDP-induced ototoxicity.

## Introduction

Since the discovery of its anticancer properties in the 1960’s, Cisplatin [*cis*-diammine dichloroplatinum (II); CDDP] has been widely used as the most potent chemotherapeutic drug for a variety of solid tumors, such as those found in testicular, ovarian, breast, head and neck, lung, and many other types of cancers (Wang and Lippard, 2005; Cepeda et al., 2007). Despite decades of research, effective approaches against CDDP-induced side effects including ototoxicity, nephrotoxicity, and neurotoxicity remain unavailable (Rybak et al., 2007; Florea and Büsselberg, 2011; Kim et al., 2018). The ototoxicity induced by CDDP limits its utility and therapeutic profile in both children and adult patients (Rybak et al., 2007; Langer et al., 2013). Therefore, finding effective medications that have a therapeutic effect on existing CDDP-induced ototoxicity remains an unmet medical need.

CDDP ototoxicity is manifested as bilateral, irreversible sensorineural hearing loss (Fang and Xiao, 2014). The ototoxicity is common, in particular among children (Li et al., 2004). The organ of Corti, the spiral ganglions, and the stria vascularis are profoundly damaged in CDDP injury (Gabaizadeh et al., 1997; Tsukasaki et al., 2000; Cardinaal et al., 2004). It’s noteworthy that the severe hair cell loss is primarily found in CDDP exposure (Wang et al., 2003; van Ruijven et al., 2005). Although CDDP accumulation is consistently high in the stria vascularis (Breglio et al., 2017), the sensory cells in the cochlea, including outer hair cells (OHCs) and inner hair cells (IHCs), are more susceptible to CDDP-induced damage (Borse et al., 2017).

Autophagy is known to be a general cellular response to starvation or stress that degrades cytoplasmic waste or aggregation depending on lysosome pathway (Klionsky et al., 2016). In addition to the maintenance of cellular homeostasis, autophagy also plays important roles in development, physiology, and pathogenesis of a variety of diseases (Mizushima et al., 2008; Janda et al., 2012; Ohsumi, 2014; He et al., 2017; Pang et al., 2017; Song et al., 2017). In various types of hearing loss, autophagy has been proven to be a protective factor for the survival of hair cells (He et al., 2017; Pang et al., 2017). However, it is also implicated in cell death processes (Shen and Codogno, 2011). In CDDP ototoxicity, it is still not well established as to whether autophagy plays a protective or destructive role (Fang and Xiao, 2014; Youn et al., 2015; Li et al., 2018; Yin et al., 2018). Furthermore, understanding how autophagy is regulated during CDDP ototoxicity also remains elusive. In view of these findings and questions, we investigated the effects of autophagy in CDDP-induced cytotoxicity. To confirm our results, several models have been employed, including the auditory cell line HEI-OC1 (Kalinec et al., 2016), the lateral line hair cell of zebrafish (Jiang et al., 2014), and the C57BL/6 mice.

Sirtuin 1 (SIRT1), the most conserved member among a family of NAD^+^-dependent protein deacetylases, has been proven to have protective effects in various common neurodegenerative disorders (Herskovits and Guarente, 2014). In our previous study, we also demonstrated that decreased SIRT1 level was correlated with age-related hair cell loss and hearing loss in C57BL/6 mice (Xiong et al., 2014, 2015), although the detailed mechanism remains elusive. Interestingly, the effect of SIRT1 in the modulation of autophagy has been proven by the fact that SIRT1 could directly deacetylating multiple autophagy-related proteins, including ATG5, ATG7 and ATG8 (Lee et al., 2008). Therefore, in the present study we aimed to explore SIRT1’s modulation of autophagy during CDDP ototoxicity.

## Materials and Methods

### Cell Culture

Since the HEI-OC1 cell line displays a variety of markers for sensory hair cells, including math1, myosin7a and prestin, it becomes a common cell line used for the elucidation of hair cell pathology. HEI-OC1 cells (kindly provided by F. Kalinec at the House Ear Institute, Los Angeles, CA, USA) were cultured in high-glucose Dulbecco’s Modified Eagle’s Medium (DMEM; Gibco BRL, Grand Island, NY, USA), supplemented with 10% fetal bovine serum (FBS; Gibco BRL, Grand Island, NY, USA) at 33°C and 10% CO_2_ in a humidified atmosphere without antibiotics. For *in vitro* cisplatin (CDDP) toxicity test, HEI-OC1 cells were exposed to CDDP at indicated concentrations for indicated hours for cell viability analysis. HEI-OC1 cells were pretreated with different agents for 24 h and then exposed to CDDP at 20 μM for 24 h.

### Materials

Cisplatin (CDDP, Selleck, S1166, Huston, TX, USA), Rapamycin (RA, Selleck, S1039, TX, USA), 3-Methyladenine (3-MA, S2767, Selleck, Huston, TX, USA), SRT1720 (SRT1720, S1129, Selleck, Huston, TX, USA). Chloroquine (CQ, C6628, Sigma-Aldrich, MO, USA), LC3-II/LC3B (#3868, Cell Signaling Technology, Boston, MA, USA), SIRT1 (#9475, Cell Signaling Technology, Boston, MA, USA), p62 (#5114, Cell Signaling Technology, Boston, MA, USA), β-actin (#4970, Cell Signaling Technology, Boston, MA, USA), p53 (#2524, Cell Signaling Technology, Boston, MA, USA), Acetyl-p53 (#2525, Cell Signaling Technology, Boston, MA, USA), Western Antibody Dilution Buffer (RM00016, ABclonal, Cambridge, UK).

### Protein Extraction and Western Blot

Images of HEI-OC1 cells treated with different reagents were captured by optical microscope. Then, the total proteins of treated cells or tissues were extracted by RIPA lysis buffer (Thermo, 89901, USA), in which proteinase inhibitor (1:100, Selleck, TX, USA) was added. After the concentration measurements by BCA assay kit (Beyotime Biotechnology, Shanghai, China), equal amounts of protein were denatured and then separated by 12% SDS-PAGE electrophoresis, followed by transfer to polyvinylidene fluoride membranes (PVDF, Millipore, Darmstadt, Germany). The membranes were blocked in 5% non-fat milk for 1 h at room temperature. After washing with TBS containing 0.05% tween 20 (TBST) three times, the membranes were incubated with related primary antibodies (1:1,000) in TBST with 5% BSA overnight. Then, they were incubated with secondary antibodies (1:5,000–1:10,000) for 1 h after three washes with TBST. Finally, the protein signals were detected by use of the ECL kit (Millipore, WBKLS0010, Darmstadt, Germany) and analyzed by ImageJ software.

### Cell Viability Assay

Cells were seeded at the density of 2,000 cells/well in a 96-well plate and allowed to attach overnight for 16 h. After treatment with or without SRT1720 (0.5 μM) or RA (0.5 μM) for 24 h, they were exposed to CDDP (20 μM) with or without 3-MA (5 mM) for another 24 h. Next, 10 μl CCK-8 reagent (Beyotime Biotechnology, Shanghai, China) was added to each well and reacted for 2 h. Absorbance at 450 nm was detected through the Multiskan MK3 microplate reader (Labsystems, USA) for cell viability.

### Transfection of Cells With Fluorescent LC3

The lentivirus containing the green fluorescent protein (GFP)-LC3 fusion gene was purchased from Hanbio (Shanghai, China). The HEI-OC1 cells were transfected with lentivirus-mediated GFP-LC3 to generate GFP-LC3-expressing cells. HEI-OC1 cells were seeded into six-well dishes (1*10^5^ cells per well) and infected with the recombinant lentivirus following the manufacturer’s instructions (a MOI of 100). After 48 h, cells were selected by culture in the presence of puromycin for 2 weeks. Cells were treated with SRT1720 (0.5 μM) or CQ (10 μM) with or without CDDP (20 μM) injury. Observation of autophagosome formation was determined after fluorescent staining by evaluating the number of GFP puncta (puncta/cell was counted).

### Assessment of Apoptosis by Flow Cytometry

Cell apoptosis was also measured by a FITC Annexin V Apoptosis Detection Kit (BD, Franklin Lakes, NJ, USA). Briefly, cells were harvested and washed twice by cold PBS solution, and resuspended with 100 μl 1× binding buffer softly. Ten microliter Annexin V and 5 μl propidium iodide (PI) were added to each group and incubated in dark room for 15 min. Approximately 10,000 cells of each group were measured by a FACS Calibur system (BD Biosciences, Franklin Lakes, NJ, USA).

### Zebrafish Breeding

Zebrafish embryos of the ET4 transgenic wildtype hair cells that are specifically labeled produced adult fish and maintained at a density of 50 embryos per 100 mm Petri dish in 28.5°C embryo medium (15.0 mM NaCl, 0.5 mM KCl, 1.0 mM CaCl_2_, 1.0 mM MgSO_4_, 0.14 mM KH_2_PO_4_, 0.06 mM Na_2_HPO_4_, and 0.5 mM NaHCO_3_).

### Lateral Line Hair Cell Counting in Zebrafish

Five days post-fertilization (dpf) zebrafish larvae were used for experiments. The experiment was set as eight groups, including control group (DMSO), CDDP group, RA group, CDDP with RA pre-treatment for 1 h, CDDP with RA group, SRT1720 group, CDDP with SRT1720 pre-treatment for 1 h and CDDP with SRT1720 group. The concentration of CDDP was 600 μM, RA 10 μM and SRT1720 5 μM. By using a 12-hole plate, with eight larvae per hole, each group set up two holes. After the CDDP exposure for 12 h and 24 h, 6 zebrafish larvae of each group were selected to fix at 4°C in 4% paraformaldehyde (PFA) for 0.5 h, flushed with PBST, then mounted in glycerin on 25 × 60 mm Non-slip off coverslips. We then determined the counts of three hair cell aggregations of lateral line hair cells in each zebrafish using a confocal microscope (Carl Zeiss, Germany) and calculated the average count of lateral line hair cells.

### Animals

Forty-eight C57BL/6 mice at the age of 7 weeks were obtained from Laboratory Animal Center, Sun Yat-sen University. After a hearing test to exclude hearing abnormal mice, the rest of them were randomly divided into six groups, a “Control” group (DMSO intraperitoneal injection), a “CDDP” group (16 mg/kg, intraperitoneal injection), two drug groups (rapamycin, 7.5 mg/kg, intraperitoneal injection; SRT1720, 100 mg/kg, intragastric administration) and two “CDDP+drug” groups. Auditory brainstem response (ABR) were tested 72 h after CDDP administration. Animal care and experimental treatment were carried out in accordance with the recommendations of Constitution of Animal Ethical and Welfare Committee (AEWC). The protocol was approved by the Animal Research Committee at Sun Yat-sen University.

### Auditory Brainstem Response

ABR measurements were performed when mice were anesthetized with intraperitoneal injection (100 mg/kg ketamine and 10 mg/kg xylazine mixture). Three needle electrodes were inserted sub-dermally at the vertex (active), under the left ear (reference), and the back (ground). The acoustic signals were generated, and the responses were processed with Tucker-Davis Technologies (TDT System III, Alachua, FL, USA) hardware and software. Ten-millisecond (ms) tone bursts with a 1 ms rise or fall time were presented at 4, 8, 16 and 32 kHz at a rate of 21.1/s. The average response to different sound intensity at 4 kHz, 8 kHz, 16 kHz and 32 kHz was collected and processed by TDT by attenuating the sound intensity 5 dB intervals from 100 dB to 0 dB. The hearing threshold was defined as the lowest stimulation dB level at which a positive wave in the evoked response trace was evident (Pang et al., 2016).

### Animal Drug Administration

Cisplatin (CDDP, Selleck, S1166, Huston, TX, USA) was dissolved in DMSO and intraperitoneally injected (16 mg/kg). Rapamycin (RA, Selleck, S1039, TX, USA) was dissolved in DMSO, and intragastric administration (7.5 mg/kg) was performed three times 24 h before and after CDDP exposure, and 1 h before CDDP exposure. SRT1720 (Selleck, S1129, Huston, TX, USA) was dissolved in normal saline (NS; 5 mg/ml) and intragastric administration (100 mg/kg) was performed three times, 12 h and 1 h before CDDP exposure and immediately after CDDP exposure.

### Tissue Preparation

After ABR recordings, the deeply anesthetized mice were decapitated, and the cochleae were taken out under microscope to fix in 4% PFA overnight at 4°C on the shakers. The cochleae were decalcified in 4% sodium ethylenediaminetetraacetic acid for 48 h, and then the osseous labyrinth, stria vascularis, spiral ligament, Reissner’s and tectorial membrane were carefully cut away under the microscopy, and the remaining basal membrane were subjected to immunofluorescent staining.

### Hair Cells Counting

Cochlear sections were incubated in 3% Triton X-100 for 45 min at room temperature on the shaker, washed with PBS three times and blocked with blocking solution (10% goat serum in PBS) for 1 h. Specimens were counterstained with 4’,6-diamidino-2-phenylindole (DAPI; 10 mg/ml, Sigma-Aldrich, MO, USA) for 10 min. The tissues were mounted on glass slides in 50% glycerol. Cochlear samples were observed and imaged with an Olympus BX63 microscope. Hair cells were counted from the apex to the base along the entire length of the cochlear epithelium. The percentage of hair cell loss in each 0.5 mm length of epithelium was plotted vs. cochlear length as a cytocochleogram.

### Statistical Analysis

All values were shown as mean ± SEM. and analyzed by one-way analysis of variance (ANOVA) with Fisher *post hoc* test or independent *t*-test. Values of *p* < 0.05 were considered statistically significant.

## Results

### SIRT1 and Autophagy Increase in CDDP Induced Cell Death in HEI-OC1 Cells

**Figure 1:**
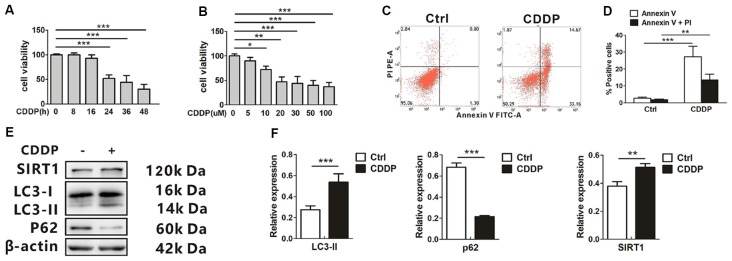
CDDP induces cell death and increases both sirtuin 1 (SIRT1) and autophagy in HEI-OC1 cells. **(A)** The CCK8 assay was performed to examine cell viability of HEI-OC1 cells in CDDP (20 μM) exposure from 0 h to 48 h (*n* = 3 individual experiments). **(B)** The CCK8 assay was performed to examine cell viability of HEI-OC1 cells in CDDP exposure from 0 μM to 100 μM for 24 h (*n* = 3 individual experiments). **(C,D)** Apoptosis measured by annexin V and propidium iodide (PI) staining for HEI-OC1 cells in CDDP (20 μM) exposure for 24 h (*n* = 3 individual experiments) and its analysis. **(E,F)** Western blots and densitometry analysis for SIRT1 and autophagy marker LC3-II and p62 in CDDP (20 μM) exposure for 24 h (*n* = 3 individual experiments). Data represent the mean ± SEM. *p < 0.05, **p < 0.01, ***p < 0.001. CDDP, cisplatin.

CDDP-induced cytotoxicity was measured by monitoring cell viability and using annexin V and PI staining in the auditory cell line HEI-OC1. In our experiments, HEI-OC1 cells started to have a decreased survival rate when exposed to 20 μM CDDP at 24 h, and this aggravated at 36 h and 48 h (Figure 1A). Then we exposed the cells to various CDDP concentrations for 24 h. Dose-dependent HEI-OC1 cell death increased by 47.0 ± 10.1% at 20 μM with a 50% of maximal effect (EC50) being ~20 μM (Figure 1B). In agreement with these findings, FACS analysis showed that CDDP induced apoptosis (Figures 1C,D). In the mRNA levels, CDDP only increased LC3B expression (Supplementary Information 1 Figure S1), but not p62. As an autophagosome marker, LC3-II increased, while the autophagic degeneration marker p62 decreased. Interestingly, CDDP significantly increased SIRT1, accompanied with autophagy (Figures 1E,F).

### Rapamycin Promotes HEI-OC1 Cell Survival After CDDP-Induced Damage

**Figure 2:**
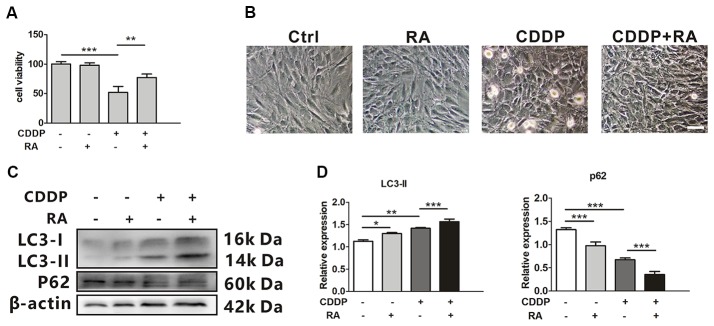
Rapamycin promotes HEI-OC1 cell survival after cisplatin-induced damage. **(A)** The CCK8 assay was performed to examine cell viability of HEI-OC1 cells following CDDP (20 μM) exposure for 24 h with or without RA (0.5 μM; *n* = 3 individual experiments). **(B)** The image of HEI-OC1 cells following CDDP (20 μM) exposure for 24 h with or without RA (0.5 μM; *n* = 3 individual experiments). **(C,D)** Western blots and densitometry analysis for autophagy marker LC3-II and p62 in CDDP (20 μM) exposure for 24 h with or without RA (0.5 μM; *n* = 3). Data represent the mean ± SEM. *p < 0.05, **p < 0.01, ***p < 0.001. CDDP, cisplatin; RA, rapamycin.

Rapamycin (RA), a well-known mTOR inhibitor, has been widely reported to induce autophagy both *in vivo* and *in vitro* (Tanemura et al., 2012). The cell death attenuated when co-treated with RA compared with the single CDDP exposure. Around 20%–30% of total cells did not experience death (Figure 2A). CDDP exposure caused more detached cells, while RA attenuated the ototoxicity (Figure 2B). Although the mRNA levels of LC3B and p62 did not change in RA treatment with or without CDDP (Supplementary Information 1 Figure S1), in the protein levels, LC3-I to LC3-II conversion further increased in RA treatment, while p62 protein decreased even in CDDP exposure (Figures 2C,D). These findings revealed that autophagy activated by RA could attenuate CDDP mediated ototoxicity.

### SIRT1 Activates Autophagy and Promotes HEI-OC1 Cells Survival After CDDP-Induced Damage

**Figure 3:**
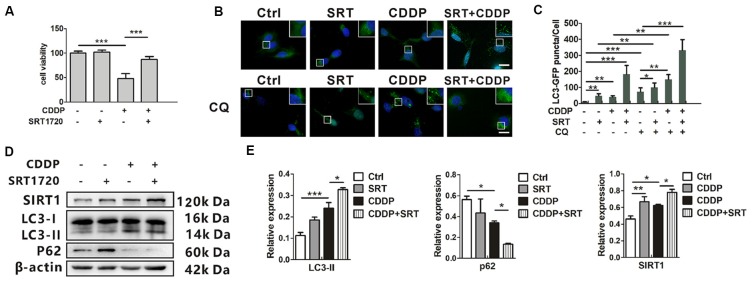
SIRT1 activates autophagy and promotes HEI-OC1 cell survival after cisplatin-induced damage. **(A)** The CCK8 assay was performed to examine cell viability of HEI-OC1 cells following CDDP (20 μM) exposure for 24 h with or without SRT1720 pre-treatment for 24 h (0.5 μM; *n* = 3 individual experiments). **(B,C)** The fluorescence image of green fluorescent protein (GFP)-LC3 HEI-OC1 cells after CDDP (20 μM) exposure with or without SRT1720 (0.5 μM) and CQ (10 μM). Scale bar, 10 μm. Quantity analysis of green puncta was detected in five cells/experiment (*n* = 3 individual experiments). **(D,E)** Western blots and densitometry analysis for SIRT1 and autophagy marker LC3-II and p62 in CDDP (20 μM) exposure for 24 h with or without SRT1720 pre-treatment for 24 h (0.5 μM; *n* = 3 individual experiments). Data represent the mean ± SEM. *p < 0.05, **p < 0.01, ***p < 0.001. CDDP, cisplatin; SRT, SRT1720.

SRT1720, a new synthetic small molecule, has been confirmed as the selective activator of SIRT1 (Yao et al., 2012; Sun et al., 2018). Previous biochemical studies have shown that the affinity of SIRT1 to SRT1720 is approximately 1,000 times as strong as that with another SIRT1 activator resveratrol. To examine the activation from SRT1720, the protein expression of p53, and ac-p53 as a deacetylated target of SIRT1, were measured by western blotting. The ratios of acetylated p53 to total p53 attenuated in HEI-OC1 cells (Supplementary Information 1 Figure S2). Then, we found that SRT1720 could reduce cell death in CDDP exposure. Around 40% of total cells were prevented from experiencing cell death (Figure 3A). In the mRNA levels, LC3B and p62 mRNA had no significant difference in SRT1720 treatment with or without CDDP exposure (Supplementary Information 1 Figure S1). To robustly demonstrate the modulation that SIRT1 activates autophagy, GFP-LC3 HEI-OC1 cells were employed to describe the modulation with autophagic degradation blocker chloroquine (CQ). CDDP or SRT1720 treatment could increase the formation of LC3, and blocker CQ captured even more. Then, the green puncta were the highest in SRT1720 treatment with CDDP exposure (Figures 3B,C). Meanwhile, SRT1720 could also further increasing LC3-II in CDDP exposure in western blot. In contrast, p62 expression decreased in CDDP exposure and further decreased with SRT1720 treatment (Figures 3D,E). These results suggest that although HEI-OC1 cells might increase SIRT1 to activate autophagy against CDDP damage that may not be enough for cells to pull through. Therefore, we find that the external activation of autophagy and SIRT1 prevent cells from death.

### SIRT1 Reduces CDDP Induced Ototoxicity Through Autophagy in HEI-OC1 Cells

**Figure 4:**
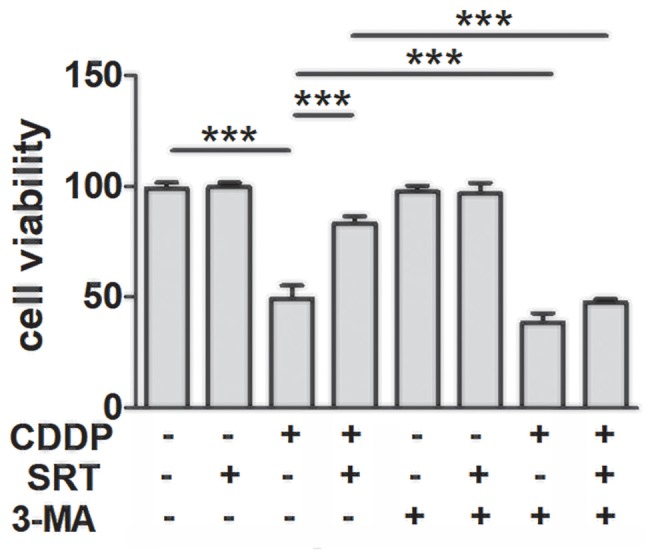
SIRT1 protects against cisplatin-induced cell death via autophagy in HEI-OC1 cells. The CCK8 assay was performed to examine cell viability of HEI-OC1 cells in CDDP (20 μM) exposure for 24 h combined with 3-MA (5 mM) treatment with or without SRT1720 pre-treatment for 24 h (0.5 μM; *n* = 4 individual experiments). Data represent the mean ± SEM. ***p < 0.001. CDDP, cisplatin; SRT, SRT1720; 3-Methyladenine, 3-MA.

To confirm whether SIRT1 reduced CDDP induced ototoxicity through autophagy, we employed the phosphatidylinositol-3-kinase inhibitor (3-MA), the most widely used autophagy inhibitor (Seglen and Gordon, 1982), to suppress autophagy. As expected, autophagy inhibition accelerated CDDP-induced cell death. Moreover, we found that the prevention of autophagy diminished the SIRT1 activation-mediated HEI-OC1 survival in the CDDP exposure (Figure 4). These results imply that the effects of SIRT1 in cell survival protection were nearly abolished in the autophagic inhibition by 3-MA. Collectively, SIRT1 reduced CDDP mediated ototoxicity via the induction of autophagy.

### Autophagy Activation Promotes the Lateral Line Hair Cell Survival After CDDP-Induced Damage in Zebrafish

**Figure 5:**
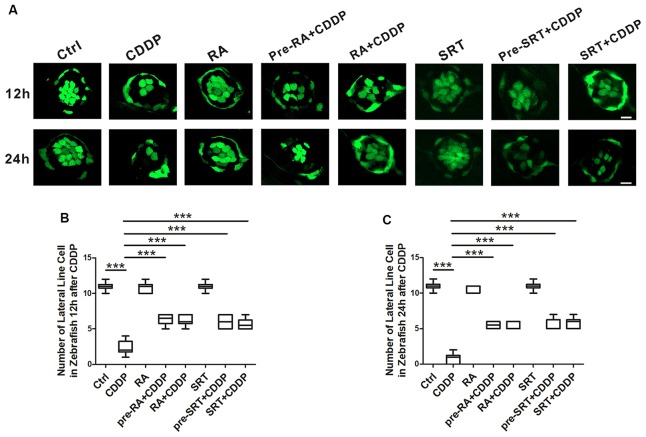
Autophagy activation promotes hair cell survival after cisplatin-induced damage in zebrafish lateral line. **(A)** Hair cell counts obtained from CDDP exposure for 12 or 24 h with or without RA or SRT1720 treatment (*n* = 6 zebrafish larvae). Scale bar, 10 μm. **(B)** The hair cells were counted at 12 h after CDDP exposure. **(C)** The hair cells were counted 24 h after CDDP exposure. Data represent the mean ± SEM. ***p < 0.001. Control, zebrafish larvae cultured in the same dose DMSO as the CDDP and RA group; RA, zebrafish larvae cultured in RA; CDDP, zebrafish larvae cultured in CDDP; pre-RA+CDDP, zebrafish larvae cultured in RA for 1 h followed by exposure to CDDP; RA + CDDP, zebrafish larvae cultured in CDDP and RA. SRT1720, zebrafish larvae cultured in SRT1720; pre-SRT1720+CDDP, zebrafish larvae cultured in SRT1720 for 1 h followed by exposure to CDDP; SRT1720 + CDDP, zebrafish larvae cultured in CDDP and SRT1720. CDDP, cisplatin; RA, rapamycin; SRT, SRT1720.

The lateral line of zebrafish can sense water motion and initiate the appropriate behavioral response for capturing prey and avoiding predators. The lateral line hair cells are located in the skin and easy to observe. Both lateral line and ear hair cells are the sensory hair cells, and they develop and differentiate by similar developmental mechanisms. Mutations in genes disrupting hair cell function in the zebrafish lateral line and vestibular system also cause deafness in humans (Nicolson, 2005). Since both the lateral line and ear hair cells share the similar genetic background, and the lateral line accessibility makes it easier in experimental manipulation and visualization, the zebrafish recently has been recognized as an excellent model for discovering and functionally characterizing genes crucial for hair cell pathology (Behra et al., 2009; Brignull et al., 2009; Liang et al., 2012; Rubel et al., 2013; Jiang et al., 2014). The analysis of hair cell survival allowed us to draw comparisons between CDDP exposure with or without RA at 12 h and 24 h (Figure 5A). At 12 h after CDDP treatment, more than half of hair cells were eliminated. As expected, nearly half of hair cells remained in the pre-treat or with RA or SRT1720 in CDDP exposure. Although at 24 h after CDDP exposure, nearly all hair cells were eliminated, while pre-treatment with RA or SRT1720 significantly attenuated cisplatin-induced hair cell loss (Figures 5A–C).

### Autophagy Activation Promotes Hair Cell Survival After CDDP-Induced Damage in C57BL/6 Mice

**Figure 6:**
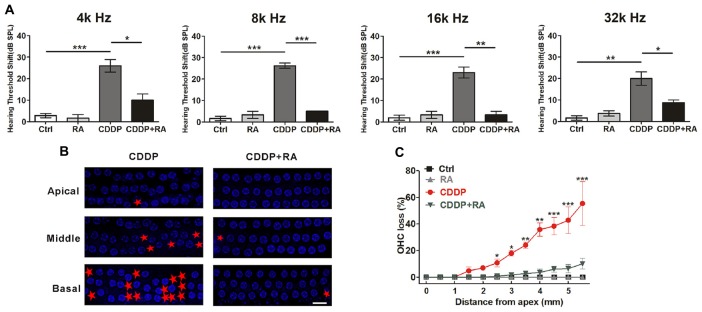
Autophagy activation attenuates cisplatin-induced hair cell loss and hearing loss in C57BL/6 mice. **(A)** Auditory brainstem response (ABR) thresholds decreased with RA (7.5 mg/kg, intraperitoneal injection pre-24 h, pre-1 h, post-24 h) treatment mice in CDDP (16 mg/kg, intraperitoneal injection) exposure compared with the CDDP groups at 4, 8, 16 and 32 kHz (*n* = 6 mice). **(B,C)** Surface preparations were stained with 4′,6-diamidino-2-phenylindole (DAPI). Hair cell counts obtained for the CDDP and CDDP+RA group (*n* = the right cochlea of six mice). Scale bar, 10 μm. Data represent the mean ± SEM. *p < 0.05, **p < 0.01, ***p < 0.001. Control, mice were intraperitoneally injected with the same dose DMSO as the CDDP group; RA, mice were intraperitoneally injected with rapamycin three times; CDDP, mice were intraperitoneally injected with CDDP; CDDP+RA, mice were intraperitoneally injected with CDDP once and rapamycin three times. CDDP, cisplatin; RA, rapamycin; Apical, the apical turn; Middle, the middle turn; Basal, the basal turn; Red asterisks, lost hair cells.

To demonstrate the major role that the activation of autophagy plays in CDDP-mediated hearing loss, we examined the effect of RA on CDDP-induced hearing loss. C57BL/6 mice at 7 weeks old developed a significant ABR threshold shift after CDDP exposure at various frequencies (26.0 ± 6.5 dB at 4 kHz, 26.3 ± 2.5 dB at 8 kHz, 23.0 ± 5.7 dB at 16 kHz and 20.0 ± 7.1 dB at 32 kHz). To supplement with RA at 20 mg/kg 24 h before and after CDDP exposure, plus 1 h before CDDP exposure, we used intraperitoneal injection. RA significantly reduced CDDP-mediated auditory threshold shifts at various frequencies (10.0 ± 5.0 dB at 4 kHz, 5.0 ± 0.0 dB at 8 kHz, 3.3 ± 2.9 dB at 16 kHz and 8.8 ± 2.5 dB at 32 kHz; Figure 6A). Additionally, RA significantly decreased OHC loss in CDDP exposure (Figures 6B,C). Taken together, these results support that RA strengthened OHCs’ survival in CDDP ototoxicity in both zebrafish and mice. Moreover, RA attenuated CDDP-mediated hearing loss in C57BL/6 mice.

### SIRT1 Activation Attenuates CDDP-Induced Hair Cell Loss and Hearing Loss in C57BL/6 Mice

**Figure 7:**
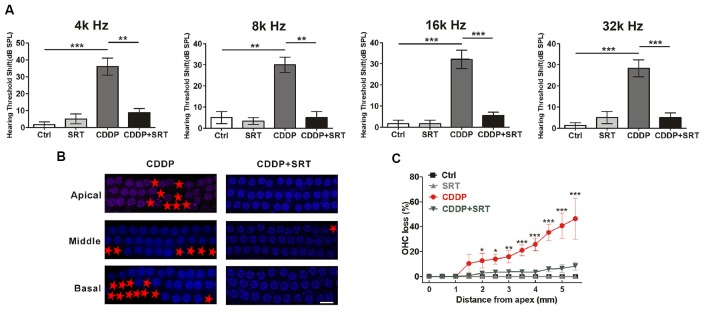
SIRT1 activation attenuates cisplatin-induced hair cell loss and hearing loss in C57BL/6 mice. **(A)** ABR thresholds decreased with SRT1720 (100 mg/kg, intragastric administration pre-12 h, pre-1 h, post-12 h) treatment mice following CDDP (16 mg/kg, intraperitoneal injection) exposure compared with the CDDP group at 4, 8, 16 and 32 kHz (*n* = 6 mice). **(B,C)** Surface preparations were stained with DAPI. Hair cell counts obtained from the CDDP and CDDP+SRT1720 group (*n* = the right cochlea of six mice). Scale bar, 10 μm. Data represent the mean ± SEM. *p < 0.05, **p < 0.01, ***p < 0.001. Control, mice intraperitoneally injected with the same dose DMSO as the CDDP group; SRT1720, mice gavage SRT1720 three times; CDDP, mice intraperitoneally injected with CDDP; CDDP + SRT1720, mice intraperitoneal injected with CDDP once and gavage SRT1720 three times. CDDP, cisplatin; SRT, SRT1720; Apical, the apical turn; Middle, the middle turn; Basal, the basal turn; Red asterisks, lost hair cells.

As proven before, SIRT1 also raised HEI-OC1 cells survival via autophagy in CDDP exposure. To demonstrate whether the activation of SIRT1 attenuates CDDP-mediated hearing loss in mice, we examined the effect of SRT1720 on CDDP-induced hearing loss. In agreement with the experiment before, CDDP developed a severe hearing loss at various frequencies (36.0 ± 11.4 dB at 4 kHz, 30.0 ± 8.9 dB at 8 kHz, 32.1 ± 11.5 dB at 16 kHz and 28.3 ± 4.0 dB at 32 kHz; Figure 7A). To supplement with SRT1720 at 100 mg/kg 12 h before and after, plus 1 h before CDDP exposure, we used gavage. SRT1720 significantly reduced CDDP-mediated auditory threshold shifts at various frequencies (8.8 ± 4.8 dB at 4 kHz, 5 ± 5 dB at 8 kHz, 5.5 ± 3.7 dB at 16 kHz and 5 ± 5 dB at 32 kHz; Figure 7A). Additionally, SRT1720 significantly decreased OHC loss in CDDP exposure (Figures 7B,C). Collectively, SRT1720 strengthens OHC survival to attenuate CDDP induced hearing loss in C57BL/6 mice.

## Discussion

Although the relationship between autophagy and cell death has been extensively investigated in the past decade, whether the activation of autophagy induces the cell death or survival in diseases is still controversial. Autophagy is known to be a general cellular process to strengthen cell ability to survive under starvation or stress conditions (Klionsky et al., 2016). However, excessive activation of autophagy leads to autophagic cell death (Yu et al., 2004; Ryter et al., 2014), such as cardiac myocyte death during ischemia/reperfusion (Liu et al., 2016). In contrast, the activation of autophagy also plays a positive role during various pathological and physiological states (Rubinsztein et al., 2015; Deng et al., 2017; Song et al., 2017), including aminoglycoside-induced and age-related hearing loss (He et al., 2017; Pang et al., 2017). Recent work has shown that the role of activation of autophagy in CDDP ototoxicity is still being debated. Autophagy, more specifically mitophagy, alleviated CDDP-induced ototoxicity (Fang and Xiao, 2014; Yang et al., 2018). In addition, the increase of the key autophagy protein Beclin-1 was observed in the model of attenuating ototoxicity induced by CDDP (Fang and Xiao, 2014; Yang et al., 2018). These results suggest that autophagy is important in preventing CDDP-induced ototoxicity. In contrast, autophagy was significantly increased after exposure to CDDP for 48 h along with cell death (Youn et al., 2015). At that moment, the autophagy inhibitor can help to decrease CDDP-induced cell apoptosis (Li et al., 2018). However, the significant apoptosis starts at 24 h, the same as our current work, and the excessive activation of autophagy occurred 48 h after CDDP exposure, which made it difficult to account for the apoptosis. Perhaps the excessive activation of autophagy along with cell death was only part of the progress at the later stage. To thoroughly determine the role of activation of autophagy at the early stage of CDDP-induced injury, when we could intervene, we employed three different models, an auditory cell line of HEI-OC1 cells, the lateral line hair cells of zebrafish, and C57BL/6 mice. We found that the induction of autophagy was markedly increased when the CDDP-induced cell death started in HEI-OC1 cells. Then, enhancing or blocking autophagy with autophagy activators or inhibitors could help us to understand the role of autophagy in CDDP ototoxicity. With the activator of autophagy rapamycin, the CDDP induced cell death was alleviated in HEI-OC1 cells, which agrees with the results in the lateral line hair cells of zebrafish. Meanwhile, the inhibitor of autophagy 3-MA promoted CDDP-induced HEI-OC1 cells death. Following CDDP exposure, the mice developed severe hearing loss, which is consistent with the results of other studies observing a 35 dB–55 dB threshold shift at 4, 8, 16 and 32 kHz (Kim et al., 2014; Benkafadar et al., 2017; Breglio et al., 2017). However, our ABR results showed no difference between different frequencies, while one study indicated higher levels of severity at the highest frequencies (Breglio et al., 2017). Furthermore, the activator of autophagy rapamycin alleviated the CDDP-maintained hair cell death in C57BL/6 mice and attenuated hearing shift. These findings suggest that autophagy plays a protective role against CDDP injury as the way to rescue itself until the point where the damage breaks through its limit.

It has been postulated that the generation of excessive ROS is considered to be one of the major causes of CDDP-induced ototoxicity, in particular the sensory cells of the organ of Corti (Kopke et al., 1997; Dehne et al., 2001; Korver et al., 2002; Hyppolito et al., 2006; Hill et al., 2008). Antioxidants showed good promise against CDDP-induced hearing loss (Borse et al., 2017). On the basis of what has been reported so far, autophagy could suppress ROS accumulation in cells by the p62 delivery pathway (Wang et al., 2018) and its specific mitophagy pathway (Kim et al., 2007). To promote cell survival, induced autophagy decreases ROS concentration and reduces the oxidative damage to biomolecules and organelles (Filomeni et al., 2015; Van Erp et al., 2017). Additionally, in neomycin or gentamicin ototoxicity, autophagy mediates its protective effects by reducing levels of ROS (He et al., 2017). Although the experimental conditions differ between reports in different tissue and our study, our data in HEI-OC1 cells, the lateral line hair cells of zebrafish and the cochlea hair cells in mice are consistent with these studies regarding the activation of autophagy against cytotoxicity, especially CDDP-induced ototoxicity.

Since autophagy activation is an important contributor to alleviate CDDP-induced ototoxicity, the underlying modulator-induced autophagy is not fully understood. SIRT1, the well-studied NAD^+^-dependent deacetylase, has been proven to have protective effects in various common neurodegenerative disorders (Herskovits and Guarente, 2014). Besides, SIRT1 can extend lifespan in lower organisms (Finkel et al., 2009; Burnett et al., 2011). According to recent studies, in addition to the FoxO-mediated mechanisms (Hariharan et al., 2010; Kume et al., 2010), SIRT1 could also directly deacetylate autophagy proteins, including ATG5, ATG7 and ATG8, to induce autophagy (Lee et al., 2008). Therefore, SIRT1 is one of the modulators of autophagy. In our previous study, we found that the hearing loss is associated with the expression of SIRT1 in the hair cells of C57BL/6 mice (Xiong et al., 2014). SIRT1 plays a protective role to prevent hair cell death in age-related hearing loss (Xiong et al., 2015). As expected, the levels of SIRT1 significantly increased in the hair cell after CDDP exposure, which was accompanied by autophagic induction in the current work. We speculated that SIRT1 might function upstream of autophagy and protect cells against hair cell toxicity and cell death during the early stage of CDDP-induced injury since the hair cells are eager to survive. By enhancing SIRT1 activity with its specific activator SRT1720, the autophagic cavity further improved. Moreover, the CDDP-induced cell death was alleviated in HEI-OC1 cells, in the lateral line hair cells of zebrafish and in cochlear hair cells of C57BL/6 mice. Nevertheless, the protective effect on cell survival was almost diminished by the autophagy inhibitor. These results reveal that the mechanism of increasing SIRT1 to activate autophagy is the way hair cells attenuate CDDP-induced cell death. To our knowledge, this is the first study that describes the modulation between SIRT1 and autophagy in CDDP exposure in hearing.

Apart from the induction of autophagy, rapamycin can modulate other cellular processes (Martin et al., 2017). We have performed immunoblot experiments to detect autophagy via the combined analysis of LC3-II and p62 levels. Rapamycin’s ability to alleviate CDDP-induced ototoxicity is at least partly due to its effect on autophagy. For further study, a specific autophagy modulator needs to be employed. In the previous studies, rapamycin and SRT1720 were applied to animals by systemic administration. However, considering that these agents might inhibit CDDP’s chemotherapeutic efficacy (Lawenda et al., 2008), local application should be considered as the way to intervene in the SIRT1/autophagy activation.

In conclusion, this study shows that CDDP injury activates SIRT1 and autophagy in HEI-OC1 cells. We report for the first time that there is a protective way for the sensory hair cells to rescue themselves by raising SIRT1 and autophagy at the early stage of CDDP injury. Both SIRT1 and autophagy play important roles in hair cell survival after CDDP exposure in HEI-OC1 cells, the lateral line hair cells of zebrafish, and in C57BL/6 mice. Our results suggest that SIRT1 modulates autophagy in CDDP ototoxicity, and provide new insights into the interplay between autophagy and CDDP-induced cell death. On the basis of the present results, we can suggest potential therapeutic strategies for overcoming the CDDP-induced ototoxicity through SIRT1 and autophagy activation.

## Author Contributions

JP conceived and designed the study, interpreted and analyzed the data, and wrote the manuscript. HX conceived and designed the study, interpreted and analyzed the data, and approved the final manuscript. TZ designed and performed the experiment, interpreted and analyzed the data, and wrote the manuscript. YZ and HY conceived and designed the study, interpreted and analyzed the data, and wrote the manuscript. GC, YY, and HC performed the experiment. HJ, ZS, HL, LL, YX, SC, QH, ML, XZ, XX, YO, and YC conceived and designed the study, interpreted and analyzed the data.

## Conflict of Interest Statement

The authors declare that the research was conducted in the absence of any commercial or financial relationships that could be construed as a potential conflict of interest.
