# Supplementary material for: Sirtuin 1 and Autophagy Attenuate Cisplatin-Induced Hair Cell Death in the Mouse Cochlea and Zebrafish Lateral Line
Source: Front Cell Neurosci. 2019 Jan 14;12:515. doi: 10.3389/fncel.2018.00515 (PMC6339946; doi:10.3389/fncel.2018.00515)
Supplement: Supplementary file 1 [file Table_1.docx]

Supplementary Information 1

**Fig. S1**


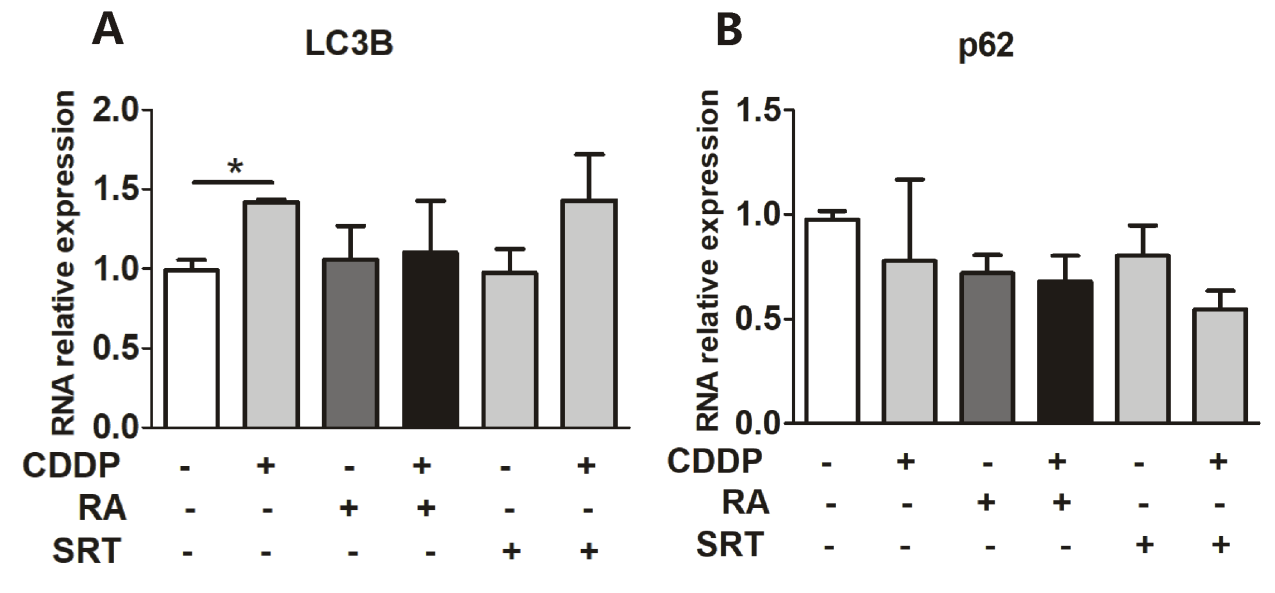


Fig. S1 The mRNA expression of LC3B and p62 in HEI-OC1 cells treated with rapamycin (0.5 μM) or SRT1720 (0.5μM) with or without CDDP (20μM) exposure. qPCR analysis for autophagy marker LC3-II (A) mRNA and p62 (B) mRNA. (n = 3 individual experiments). Data represent the mean ± SEM. * *p* < 0.05. CDDP, cisplatin; SRT, SRT1720; RA, rapamycin.

**Fig. S2**


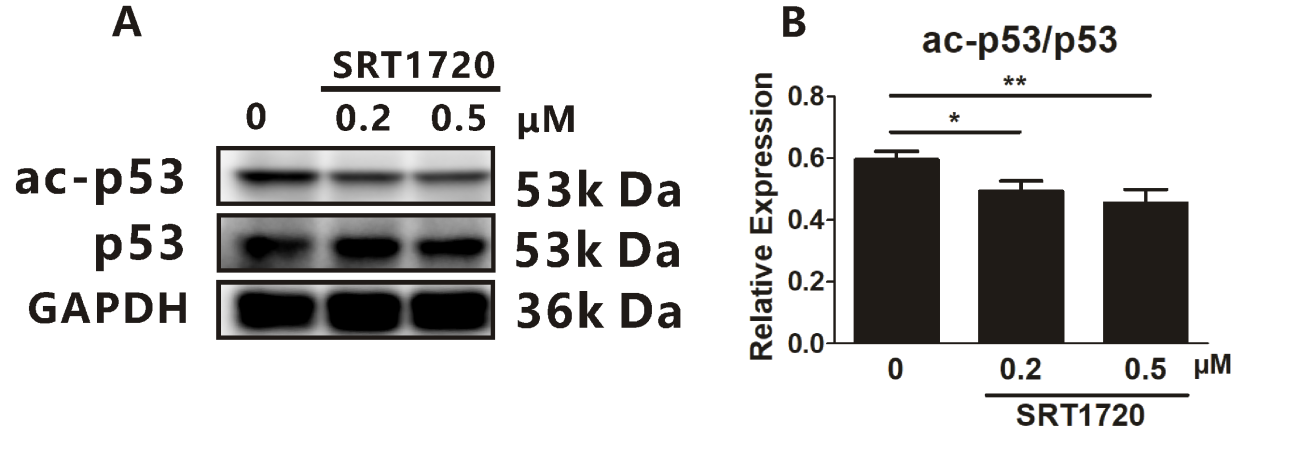


Fig. S2 (A)Western blots of acetylated and total p53 with SIRT1 activator SRT1720 (0.5μM), and (B) quantification of the acetylated p53/total p53 (n = 3 individual experiments). Data represent the mean ± SEM. * *p* < 0.05, ** *p* < 0.01. ac-p53, acetylated p53; SRT, SRT1720.
