# Supplementary material for: Sirtuin 1 and Autophagy Attenuate Cisplatin-Induced Hair Cell Death in the Mouse Cochlea and Zebrafish Lateral Line
Source: Front Cell Neurosci. 2019 Jan 14;12:515. doi: 10.3389/fncel.2018.00515 (PMC6339946; doi:10.3389/fncel.2018.00515)
Supplement: Supplementary file 2 [file Table_2.docx]

Supplementary Information 2

The dosage of rapamycin, SRT1720, 3-MA and CQ in HEI-OC1 cells.


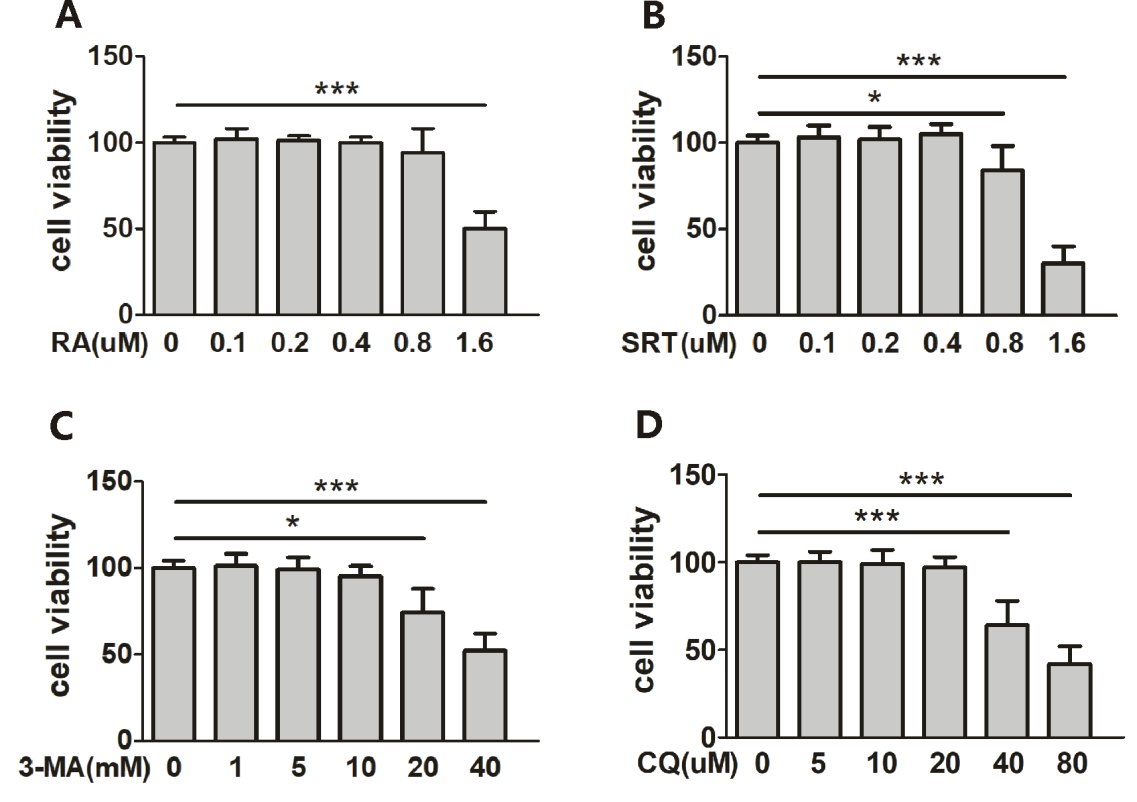


**Fig. S3** Cell viability of HEI-OC1 administrated with different reagents. (A) The CCK8 assay was performed to examine cell viability of HEI-OC1 cells treated with various concentration of rapamycin for 24 h (n = 3 individual experiments). (B) The CCK8 assay was performed to examine cell viability of HEI-OC1 cells in SRT1720 exposure from 0 to 1.6 μM for 24 h and drug-free for 24 h (n = 3 individual experiments). (C) The CCK8 assay was performed to examine cell viability of HEI-OC1 cells in 3-MA from a sequential dosage exposure for 24 h (n = 3 individual experiments). (D) The CCK8 assay was performed to examine cell viability of HEI-OC1 cells in CQ from a sequential dosage exposure for 24 h (n = 3 individual experiments). Data represent the mean ± SEM. * *p* < 0.05, *** *p* < 0.001. CDDP, cisplatin. RA, rapamycin. SRT, SRT1720; 3-MA, 3-Methyladenine. CQ, Chloroquine.

The scans of the full gel images.

WB from Fig.1


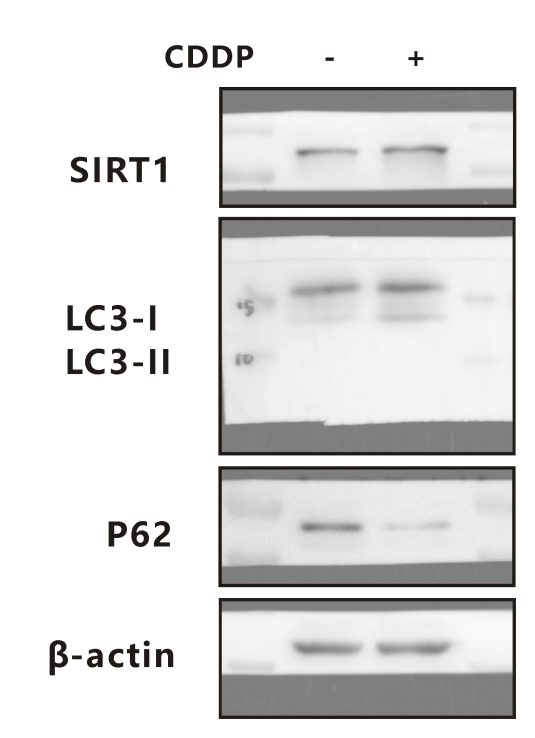


**Fig. S4** The scans of the full gel images of western blots analysis for SIRT1 and autophagy marker LC3-II and p62 in CDDP (20 μM) exposure for 24 h.

WB from Fig.2


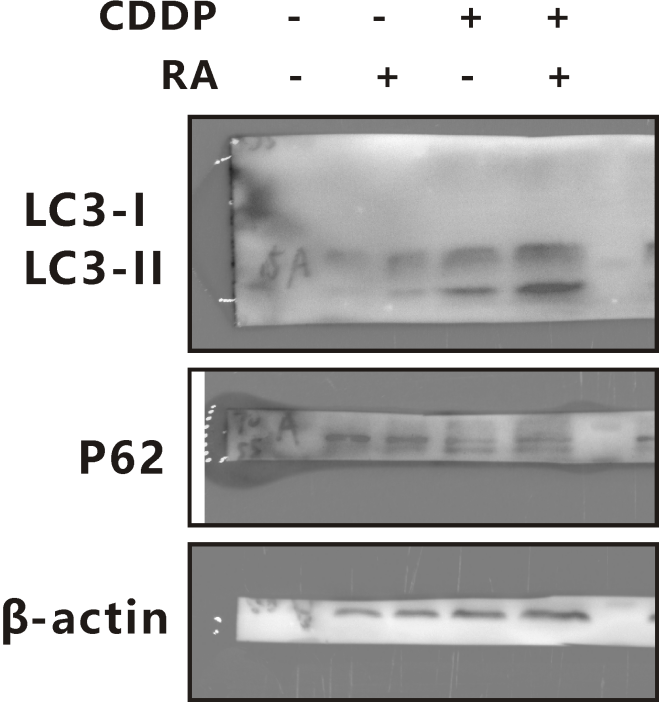


**Fig. S5** The scans of the full gel images of western blots analysis for autophagy marker LC3-II and p62 in CDDP (20 μM) exposure for 24 h with or without RA (0.5 μM).

WB from Fig.3


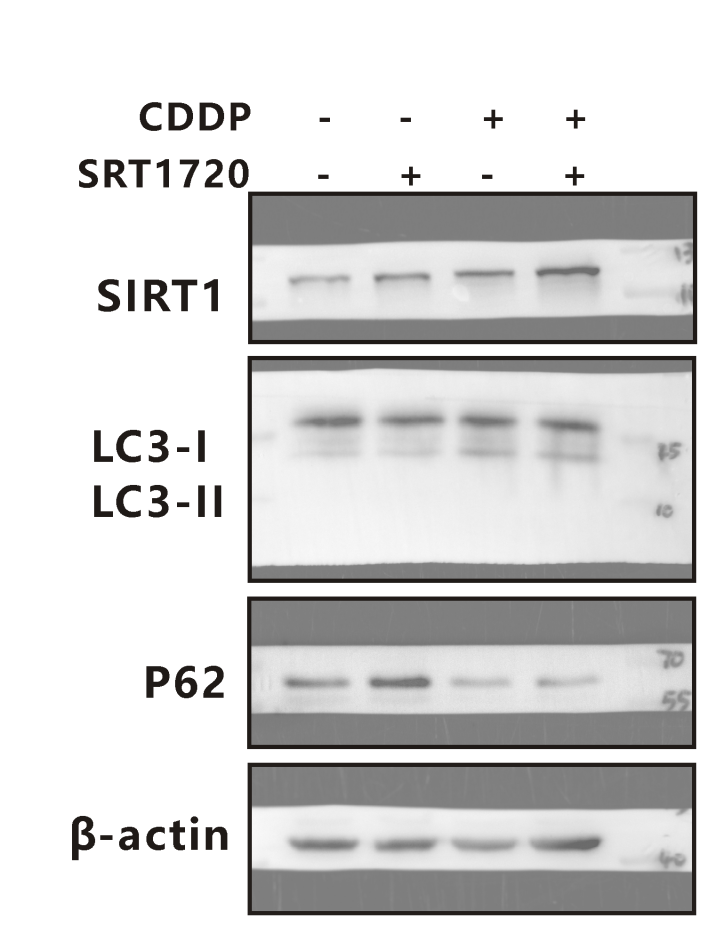


**Fig. S6** The scans of the full gel images of western blots analysis for SIRT1 and autophagy marker LC3-II and p62 in CDDP (20 μM) exposure for 24 h with or without SRT1720 pre-treatment for 24 h (0.5 μM).

WB from Fig.S2


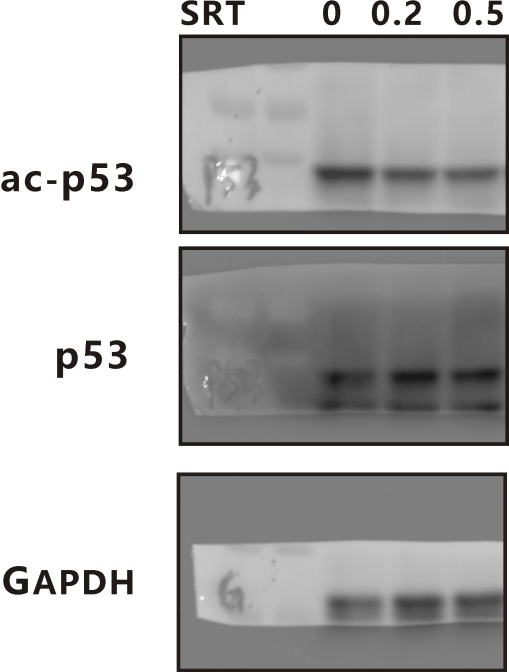


**Fig. S7** The scans of the full gel images of western blots analysis of acetylated and total p53 with SIRT1 activator SRT1720 (0.5μM)
